# Impact of Asthma on the Development of Coronary Vasospastic Angina: A Population-Based Cohort Study

**DOI:** 10.1097/MD.0000000000001880

**Published:** 2015-10-23

**Authors:** Ming-Jui Hung, Chun-Tai Mao, Ming-Yow Hung, Tien-Hsing Chen

**Affiliations:** From the Division of Cardiology, Department of Medicine, Chang Gung Memorial Hospital, Keelung, Chang Gung University College of Medicine, Keelung City, Taiwan (MJH, CTM, THC); Division of Cardiology, Department of Internal Medicine, Shuang Ho Hospital, Taipei Medical University, New Taipei City, Taiwan (MYH); Department of Internal Medicine, School of Medicine, College of Medicine, Taipei Medical University, Taipei City, Taiwan (MYH); and Graduate Institute of Clinical Medical Sciences, Chang Gung University College of Medicine, Taoyuan, Taiwan (MYH).

## Abstract

Although asthma increases the risk of cardiovascular disease, little is known about the relation of asthma and its severity to coronary vasospastic angina (CVsA). We hypothesized that asthma contributed to the development of CVsA.

Patients in this population-based cohort study were retrospectively collected from the Taiwan National Health Insurance database. Using propensity score matching, subjects were stratified at a 1 : 4 ratio into a study group comprising 3087 patients with a diagnosis of CVsA, and a control group consisting of 12,348 patients who underwent coronary intervention for obstructive coronary artery disease (CAD) during the period 2000 to 2011.

Asthma significantly increased the risk of new-onset CVsA independent of other comorbidities [adjusted odds ratio (OR) = 1.85, 95% confidence interval (95% CI) = 1.47–2.32, *P* < 0.001]. In addition, the risk of new-onset CVsA was significantly higher in previous users of oral or inhaled corticosteroids (oral corticosteroids: OR = 1.22, 95% CI = 1.01–1.49, *P* = 0.04; inhaled corticosteroids: OR = 1.89, 95% CI = 1.28–2.79, *P* = 0.001). In addition, the prevalence of asthma was highest among patients with CVsA alone, followed by patients with CAD and CVsA and patients who underwent coronary intervention for CAD alone (*P* trend < 0.001).

Our study suggests that asthma is independently associated with CVsA and prior steroid use increases the risk of CVsA development.

## INTRODUCTION

Coronary vasospastic angina (CVsA), a condition characterized by the presence of reversible stenosis of an epicardial coronary artery, is an important cause of ischemic heart disease and sudden cardiac death.^1,2^ The precise mechanisms governing the development of CVsA have not been definitively elucidated. In our previous studies, we found that patients with CVsA have abnormally high levels of inflammatory markers, such as high-sensitivity C-reactive protein (hs-CRP), indicating that inflammation is associated with CVsA.^3–5^ Other investigators have also shown that inflammation plays an important role in coronary spasm and variant angina.^6,7^ Although clinical similarities exist between CVsA and atherosclerotic coronary artery disease (CAD), the risk factors for those cardiac conditions are not same. For example, known risk factors for CAD include hypertension, diabetes mellitus, dyslipidemia, and cigarette smoking, whereas only cigarette smoking has been identified as a major clinical risk factor for CVsA.^8^ It has also been shown that treatment with calcium antagonist/nitrates and cessation of smoking can achieve favorable outcomes for patients with CVsA who experienced cardiac arrest.^9^ Interestingly, treatment with calcium antagonist/nitrates has also been shown to be associated with a reduction in levels of the inflammatory marker hs-CRP.^10^

Asthma increases the risk of cardiovascular disease,^11^ and studies have shown that systemic inflammation contributes to the high rates of cardiovascular complications in patients with asthma, such as atherothrombosis.^12,13^ Although the pathogenesis of atherothrombosis is complex, low-grade systemic inflammation is one of the central promoters of plaque and clot formation. The inflammatory cytokines interleukin-6 and interleukin-17 have been shown to be related to the severity of asthma.^14^ Interestingly, we also found that interleukin-6 levels are independently associated with CVsA.^15^ In a recent in vitro study,^16^ we demonstrated that increased level of interleukin-6 was associated with a decrease in endothelial nitric oxide synthase activation and subsequently a decrease in nitric oxide bioavailability. In a retrospective chart review, Prinzmetal's angina was shown to be associated with obstructive lung disease.^17^ The reversible airway obstruction of asthma and reversible coronary artery obstruction of coronary vasospasm may share similar pathophysiologies, for example, smooth muscle contraction and inflammation. In this population-based cohort study, we investigated whether asthma contributes to the development of CVsA.

## PATIENTS AND METHODS

### Data Source

Patients in this population-based cohort study were retrospectively collected from the Taiwan National Health Insurance (NHI) Research Database. The International Classification of Diseases, Ninth Revision, Clinical Modification [ICD-9-CM] codes for clinical diagnosis were used in the NHI database, which collect information of more than 23 million people, representing more than 98% of Taiwan residents.^18^ This well-defined health insurance database has been described in more detail in previous studies.^19,20^ The encryption system used in the NHI Research Database makes identifying individuals impossible. Confidentiality assurances were addressed by abiding by the data regulations of the Bureaus of NHI, and institutional review board approval was waived.

### Patient Identification

The study group comprised patients ≥18 years of age with newly diagnosed CVsA (ICD-9-CM code 4131) at index hospitalization during the period January 1, 2000 to December 31, 2011. Similar to our prior studies,^4,5,8,10,15^ diagnostic criteria of CVsA were angina at rest associated with electrocardiographic ST-segment elevation or depression, which was relieved by sublingual nitroglycerin; without obstructive CAD on coronary angiography after intracoronary nitroglycerin administration; and a positive intracoronary methylergonovine provocation testing result. Patients with CVsA who had received coronary intervention during index hospitalization were excluded. The control group comprised patients ≥18 years of age without CVsA who received coronary intervention for obstructive CAD (ICD-9-CM code 410.xx–414.xx except for 4131) during index hospitalization.

### Asthma and Associated Medications

A diagnosis of asthma (ICD-9-CM code 493.xx) before the index hospitalization in all study and control participants was carefully evaluated. The pharmacological treatment for asthma before index hospitalization was evaluated as follows: exposed to corticosteroids: corticosteroid use by either oral, parenteral, or inhaled within 3 years before the index hospitalization; nonexposed: all others; and nonsteriod bronchodilator use. Exposed to corticosteroids was further classified as never; new: first use within 6 months before index hospitalization; and previous: use within 7 to 36 months before index hospitalization.

### Propensity Score Analysis

To simulate a randomized trial, we used propensity score analysis (PSA) to reduce potential confounding and selection bias.^21^ The propensity score for each patient was estimated by modeling the probability of having CVsA on the basis of logistic regression. The covariates included age, sex, history of myocardial infarction (ICD-9-CM codes 410.xx and 412.xx), heart failure (ICD-9-CM codes 428.xx), stroke (ICD-9-CM codes 430.xx to 437.xx), peripheral artery disease (ICD-9-CM codes 440.0, 440.2x, 440.3x, 440.4, 440.9, 443.9, 444.2, 444.22, 444.8, 444.81, 445.0, 445.02, 250.7x, 707.1x), history of other comorbidities such as hypertension (ICD-9-CM codes 401.xx to 405.xx), dyslipidemia (ICD-9-CM codes 272.xx), diabetes mellitus (ICD-9-CM codes 250.xx), chronic kidney disease and dialysis (ICD-9-CM codes 585, 58001C,58027C), atrial fibrillation (ICD-9-CM codes 427.31), gout (ICD-9-CM codes 274.xx), malignancy (ICD-9-CM codes 140.xx to 208.xx), as well as year of the index admission. After deriving the propensity scores, each patient in the CVsA group was matched with 4 counterparts in the control group. In order to increase the power of the statistical analysis, we randomly selected a ratio of 1 : 4 (CVsA group: comparison group). The PSA algorithm was based on the nearest-neighbor method (Greedy's matching) and used the caliper radius (set as 0.5 sigma), which signifies the tolerance level for the maximum distance in the propensity score. The matching procedure was performed with the software package SAS (Version 9.3, SAS Institute, Cary, NC).

### Statistical Analysis

Categorical data are expressed as frequencies and percentages and were compared between the study and control groups using the Chi-square test. Continuous data are presented as the mean and standard deviation and were compared between the study and control groups using the independent sample *t* test. Due to the retrospective nature of case–control studies, conditional logistic regression analyses^22^ were performed to evaluate the association between asthma, the use of corticosteroids or bronchodilators, and the risk of CVsA. To rule out confounding factors, the regression analyses were adjusted for gender, age, and all of the underlying diseases listed in Table 1. In addition to evaluating the prevalence of asthma across different study groups (ie, CAD with coronary intervention, CAD with CVsA, and CVsA alone) before PSA matching, linear contrasts in the general linear model were tested (known as *P* for linear trend). All data analyses were performed with the statistical package SPSS for Windows (Version 22; SPSS Inc, Chicago, IL).

**TABLE 1:**
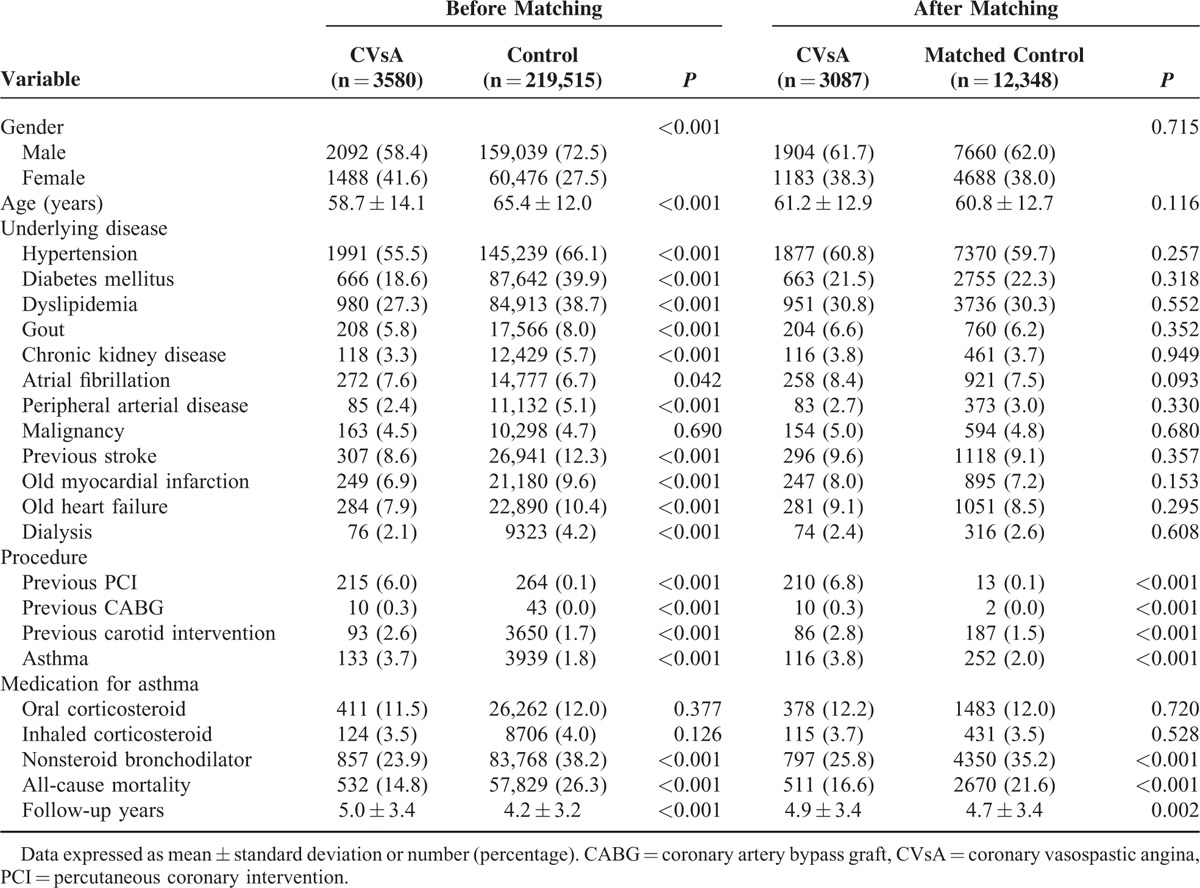
Baseline Characteristics Before and After Propensity Score Matching

## RESULTS

### Patient Characteristics

**FIGURE 1:**
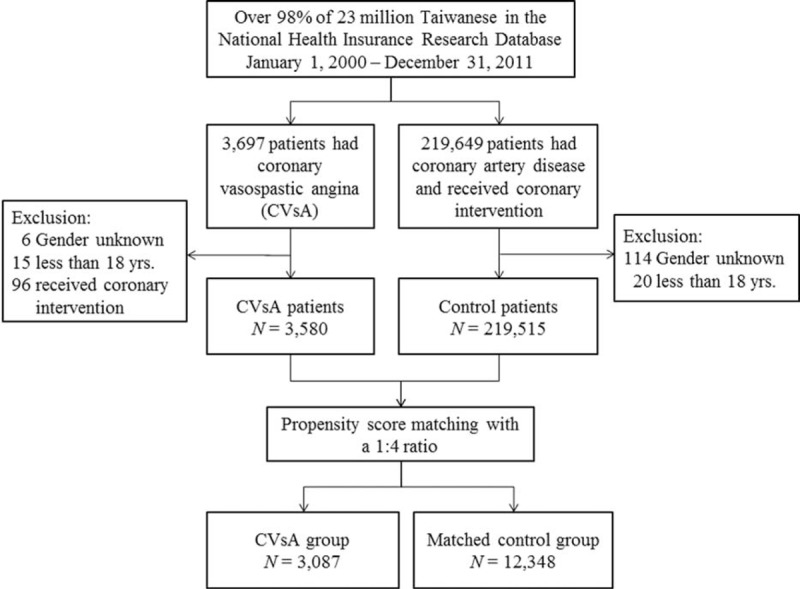
The enrollment of study patients. CVsA = coronary vasospastic angina, N = number.

During the period January 1, 2000 to December 31, 2011, a total of 3697 patients received a diagnosis of CVsA. Of them, we excluded 96 patients because they had received coronary intervention during the index hospitalization, 15 patients because they were less than 18 years old, and 6 patients because the gender was unknown. Therefore, the total number of patients in the study group before PSA matching was 3580. Also during the study period, we identified 219,649 patients who received a diagnosis of obstructive CAD and received coronary intervention. Of them, we excluded 114 patients because of unknown gender and 20 patients because they were less than 18 years old. Therefore, the control group before PSA matching consisted of 219,515 patients (Fig. 1).

Table 1 demonstrates the baseline patient characteristics before and after PSA matching. Before PSA matching, patients with CVsA were younger and more likely to be female than patients in the control group. With the exception of atrial fibrillation and malignancy, the prevalence of underlying diseases in the CVsA group was lower than that in the control group. After PSA, 3087 patients in the CVsA group were successfully matched by propensity score to 12,348 patients in the control group at a 1 : 4 ratio (Fig. 1). The distribution of clinical characteristics between the CVsA group and control group was similar after PSA matching with the exception of the higher prevalence of previous procedures and better prognosis in patients with CVsA. In both groups, the mean age was about 61 years and approximately 62% of patients were male. Bivariate analysis revealed that the prevalence of asthma was higher in patients with CVsA than in patients in the control group (3.8% vs 2.0%, *P* < 0.001).

### Evaluation of Risk for CVsA

Before adjustment, asthma was significantly associated with a higher risk of new onset CVsA [odds ratio (OR), 1.90; 95% confidence interval (CI), 1.52–2.38]. Asthma was still independently associated with an increased risk of new onset CVsA after adjustment for age and gender (OR, 1.88; 95% CI, 1.50–2.36; in model 2) and in the fully adjusted model (OR, 1.85; 95% CI, 1.47–2.32; in model 3) (Table 2).

**TABLE 2:**
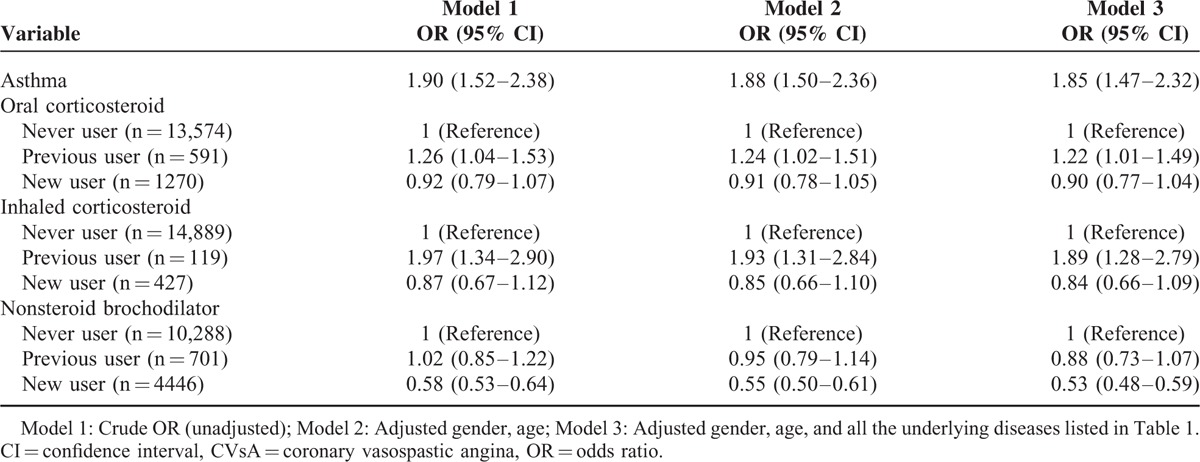
Association Between Asthma and Related Medications and Risk of CVsA

**FIGURE 2:**
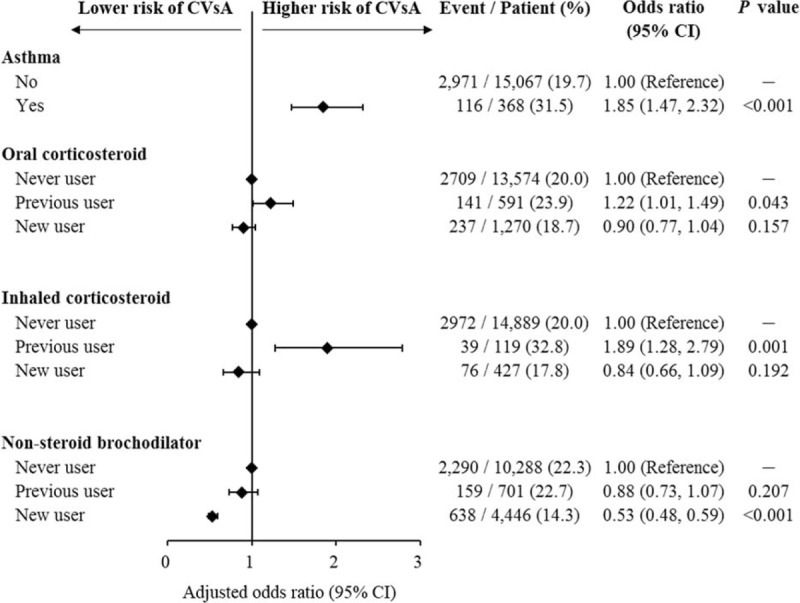
Association between asthma and related medications and risk of CVsA. CVsA = coronary vasospastic angina.

The effect of medications including corticosteroids and bronchodilators was investigated. The risk of new onset CVsA for previous users of oral corticosteroids (OR: 1.22; 95% CI: 1.01–1.49) or inhaled corticosteroids (OR: 1.89; 95% CI: 1.28–2.79) was higher than that for never users in the fully adjusted model. However, use of oral or inhaled corticosteroids within 6 months of the index date was not associated with risk of CVsA. Interestingly, new users of nonsteroid bronchodilators were less likely than never users to develop CVsA in the fully adjusted model (OR: 0.53; 95% CI: 0.48–0.59) (Fig. 2).

**FIGURE 3:**
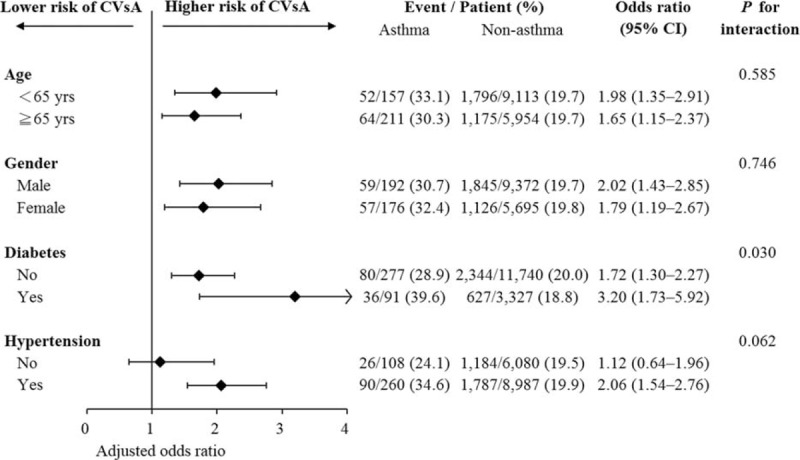
Association between asthma and risk of CVsA among predefined subgroups in terms of age, gender, diabetes, and hypertension. CVsA = coronary vasospastic angina.

To further investigate the effects of asthma on CVsA, the patient groups were stratified by age, gender, diabetes mellitus, and hypertension. We found that the effect of asthma on risk of CVsA was stronger in patients with diabetes mellitus than in patients without diabetes mellitus (*P* for interaction = 0.03) (Fig. 3).

**FIGURE 4:**
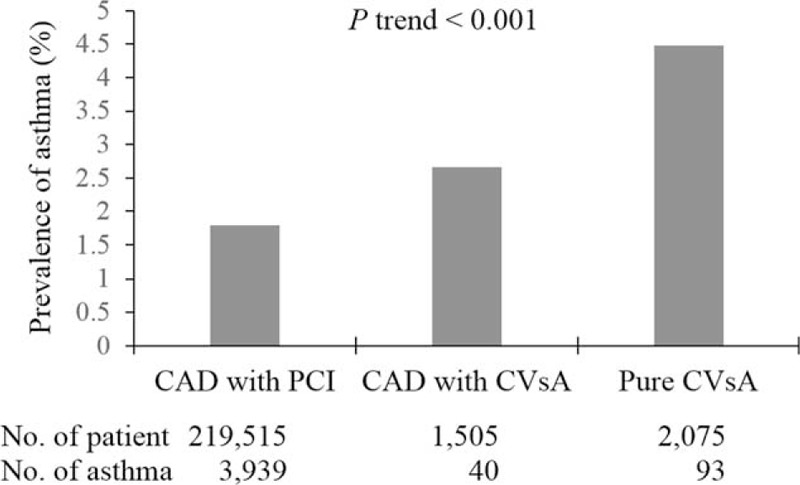
The prevalence of asthma in each study group before matching. CAD = coronary artery disease, CVsA = coronary vasospastic angina, PCI = percutaneous coronary intervention.

In addition, we also found that the prevalence of asthma was highest among patients with CVsA alone, followed by patients with CAD and CVsA and patients who underwent coronary intervention for CAD alone (*P* trend < 0.001) (Fig. 4).

## DISCUSSION

To the best of our knowledge, this is the largest observational study to demonstrate that asthma is independently associated with CVsA. We found that CVsA patients were likely to be younger and less likely to have cardiovascular risk factors and diseases than patients with obstructive CAD. In addition, less all-cause mortality was observed in CVsA patients. We also found that the prevalence of asthma was highest among patients with CVsA alone, followed by patients with CAD and CVsA and patients who underwent coronary intervention for CAD alone. Our findings are consistent with those reported by other investigators^8,23–25^ and indicate that CVsA and obstructive CAD differ in their respective pathogenesis, although they are both manifestations of ischemic heart disease. The most probable mechanisms governing the development of CVsA are vascular smooth muscle cell hypercontraction and endothelial dysfunction.^26^ Both mechanisms are linked to inflammation,^4,27,28^ which is present not only in cardiovascular disease but also in obstructive lung disease. Asthma and obstructive CAD share some risk factors, such as smoking and hypertension and both have similar pathogenic mechanisms, such as systemic inflammation. Nonetheless, the National Health and Nutrition Examination Survey found that impaired lung function is an independent risk factor for cardiovascular death, predominantly due to atherosclerotic disease.^29^ It has also been suggested that there is an important interplay between systemic inflammation and airflow obstruction in the development of ischemic heart disease.^30^

We found that a stronger association exists between asthma and CVsA than between asthma and obstructive CAD. The coexistence of CVsA with asthma has been previously reported.^31^ Larsen et al^32^ studied patients with and without obstructive CAD who had myocardial infarctions. In the majority of patients without CAD, myocardial infarction was presumably due to coronary vasospasm. They found that patients without obstructive CAD were younger than those with CAD and were more likely to have chronic lung disease, which was referred to as asthma because of the young age of the patients. In a chart review, it was found that preexisting chronic obstructive lung disease, usually asthma, was present in 36% of patients with CVsA patients, which was higher than the 5% of Americans who self-reported having asthma in the 1992 Centers for Disease Control survey.^17^ These observations suggest that there is a connection between CVsA and asthma. The shared pathphysiology might be responsible for the connection. Autonomic dysfunction appears to be a contributing factor in both disorders.^31^ Histamine provokes bronchospasm and ccoronary vasospasm, and allergic hypersensitivity reactions trigger asthmatic exacerbations and coronary vasospasm. Both disorders have significant inflammatory components.^8,33^

Previous studies of asthma found that current users of corticosteroids, especially high-dose users, were at an increased risk of developing atrial fibrillation, especially when inhaled corticosteroids were used.^34,35^ However, Huerta et al^36^ found no association between inhaled corticosteroids and cardiac arrhythmias. On the contrary, we observed that previous users, but not new users, of corticosteroids were at an increased risk of CVsA development. A possible explanation for that finding is that previous corticosteroid users have more severe inflammation, which might easily induce CVsA than new corticosteroid users. To the best of our knowledge, no studies have specifically addressed this relationship. The current study also observed that new users of noncorticosteroid bronchodilators, but not previous users, had a decreased risk of developing CVsA. Bronchodilators may stimulate and increase sympathetic activity and subsequently induce vasospasm. New users of noncorticosteroid bronchodilators might therefore be less susceptible. Further large-scale prospective studies are needed to confirm these associations.

We also observed that patients with asthma and associated diabetes mellitus had a higher risk of CVsA than patients with asthma but without diabetes mellitus. Inflammation is common in both diabetes mellitus and asthma.^13,37^ Our previous study showed that diabetes mellitus contributes to CVsA development in men with low C-reactive protein levels, but not in women.^5^ There are negative effects of diabetes mellitus and hypertension on CVsA development in patients with high C-reactive protein levels, especially in women. Although no studies have specifically addressed the relationship among CVsA, asthma, and diabetes mellitus, it is suggested that there is an interplay between systemic inflammation with asthma and diabetes mellitus in the development of CVsA. In the current study, the association between asthma and CVsA was independent of age, gender, and hypertension.

## LIMITATIONS

There are some limitations in this study. First, diagnoses of asthma and CVsA were based on ICD-9-CM codes rather than obtained through biochemical testing. To ensure the validity of the diagnoses, we selected patients who had at least 2 consecutive diagnoses by clinicians. Second, data on some variables such as smoking and body mass index, which might be related to CVsA and asthma, are not available in the NHI database. The prevalence of smoking recorded in Taiwan NHI database should be underestimated and not be reliable. In addition, smoking is also the major risk factor for the obstructive CAD. Our prior study showed that the prevalence rate of smoking is 53%, 41%, 21% in obstructive CAD, CVsA, and nonobstructive CAD, respectively.^38^ Smoking was independently associated with CVsA as compared with nonobstructive CAD. Compared with obstructive CAD group, smoking was less prevalent in CVsA. Our current study tried to investigate whether asthma contributes to the development of CVsA as compared with obstructive CAD. The effect of smoking may be considered as equal to both groups. Third, the dosage and frequency of using bronchodilators cannot be clearly identified due to the limitation of Taiwan NHI Research Database. The heart rate changed after using this medication was not obtained because heart rate was not recorded in NHI Research Database. The heart rate, which can affect outcomes of CAD,^39^ is not the major risk factor to affect the prevalence of CAD, however. Fourth, the data resource is from an insurance data, that is, Taiwan NHI Research Database, which might be less confident in data validity. As with all observational studies, nonexperimental results cannot prove causality. The value of large national databases depends on the novel hypothesis, reasonable study design, and careful analysis of the studies by researchers. Using Taiwan NHI Research Database, some researchers discovered many important medical findings.^19,20,35,40,41^ Recently, Hsing et al^42^ commented on Taiwan NHI Research Database and supported its studies, as its extensiveness and its ability to answer a wide variety of questions. Finally, the data of asthma and angina severity were not available in Taiwan NHI Research Database. Therefore, we cannot analyze the severity association between asthma and angina.

## CONCLUSION

Asthma is independently associated with CVsA and prior steroid use increases the risk of CVsA development. Large-scale prospective studies or meta-analyses are necessary to confirm our findings.
